# Supplementary material for: Mechanistic Insights into Anti-Melanogenic Effects of Fisetin: PKCα-Induced β-Catenin Degradation, ERK/MITF Inhibition, and Direct Tyrosinase Suppression
Source: Int J Mol Sci. 2025 Dec 4;26(23):11739. doi: 10.3390/ijms262311739 (PMC12692049; doi:10.3390/ijms262311739)

Supplementay information

Mechanistic Insights into Anti-Melanogenic Effects of Fisetin: PKCα-induced β-Catenin Degradation, ERK/MITF Inhibition, and Direct Tyrosinase Suppression

Zin Zin Ei [1,2], Satapat Racha [1,2,3], Hongbin Zou [4] and Pithi Chanvorachote [1,2,5,6,*]

Figure S1. Uncropped gel images for Figure 2D

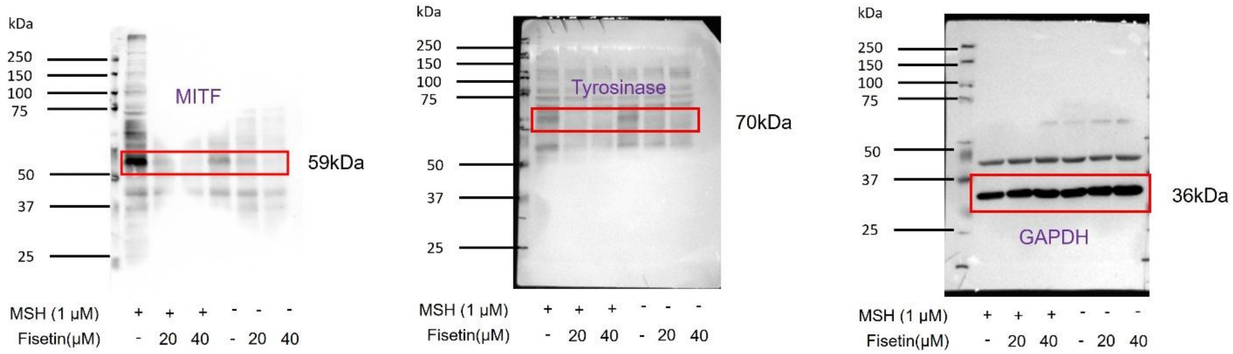

Figure S2. Uncropped gel images for Figure 3B

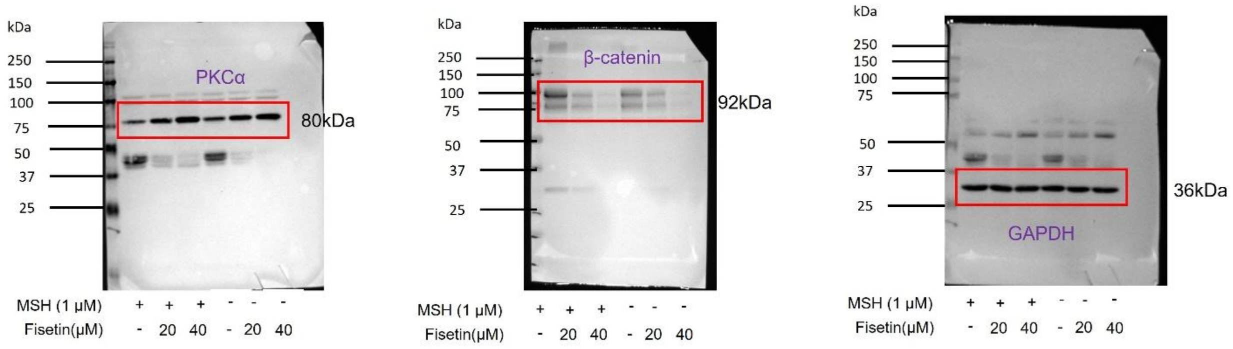

Figure S3. Uncropped gel images for Figure 3C

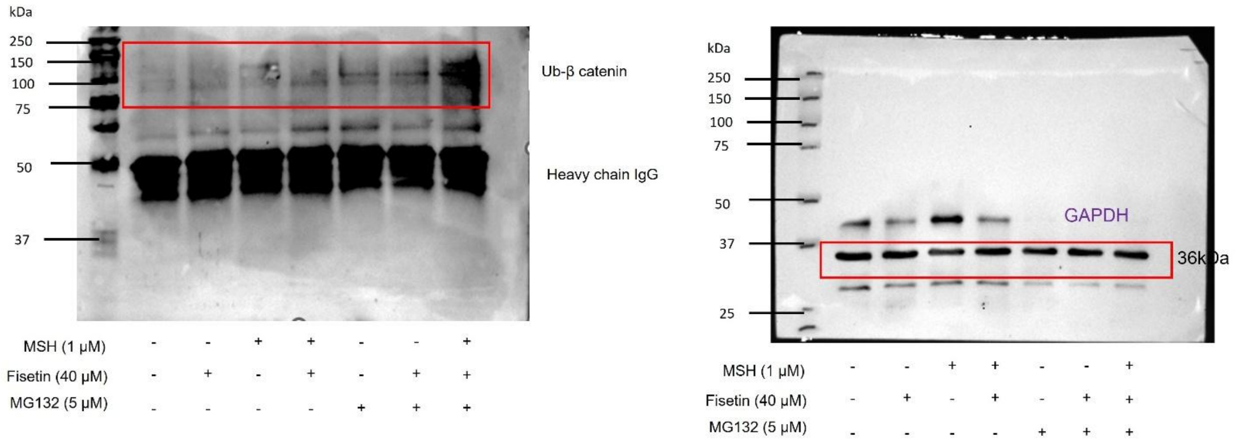

Figure S4. Uncropped gel images for Figure 4B

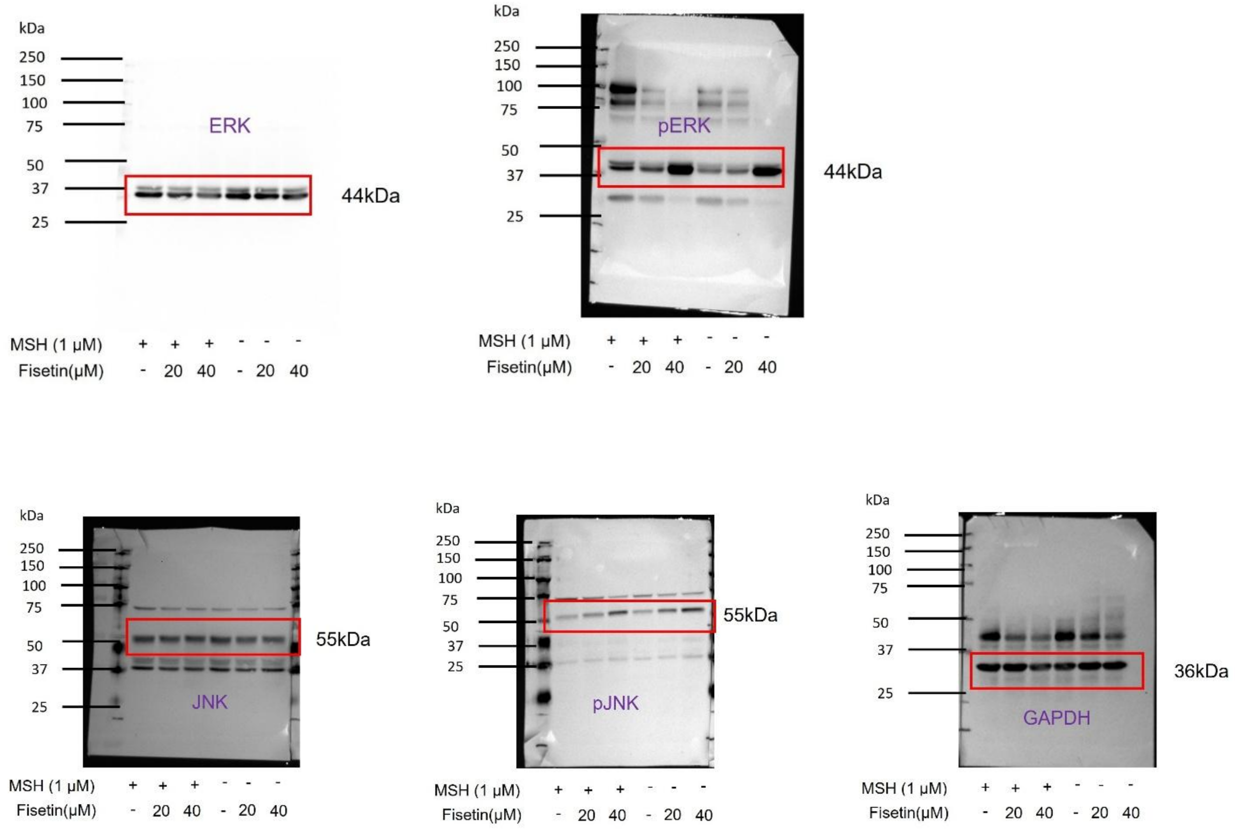

Figure S5. Uncropped gel images for Figure 4C

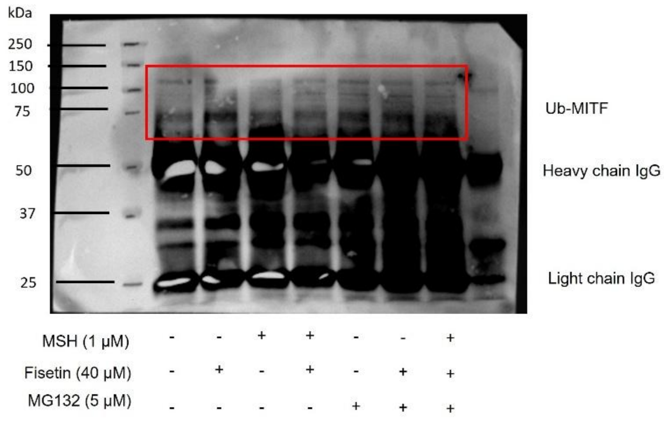

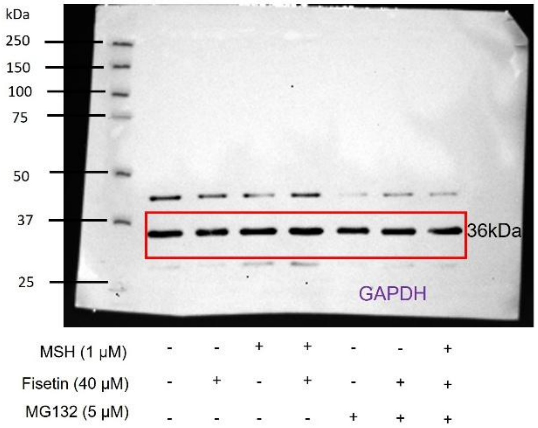

Figure S6. Uncropped gel images for Figure 4F

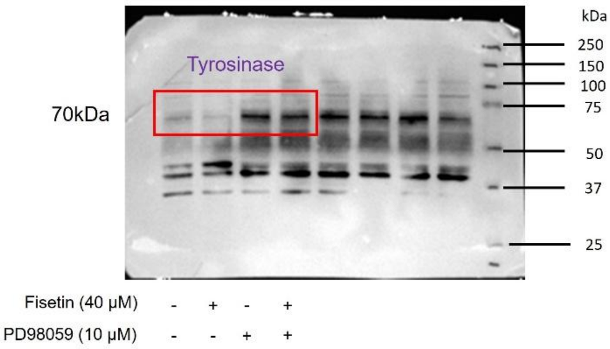

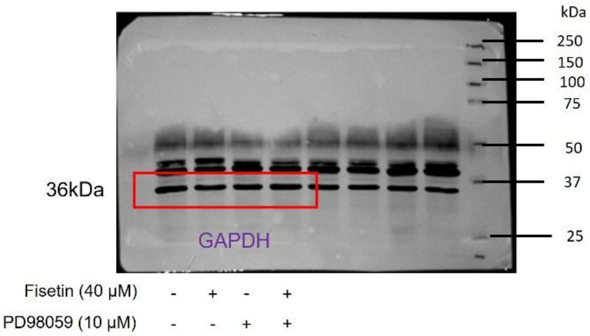

Figure S7. Uncropped gel images for Figure 4G

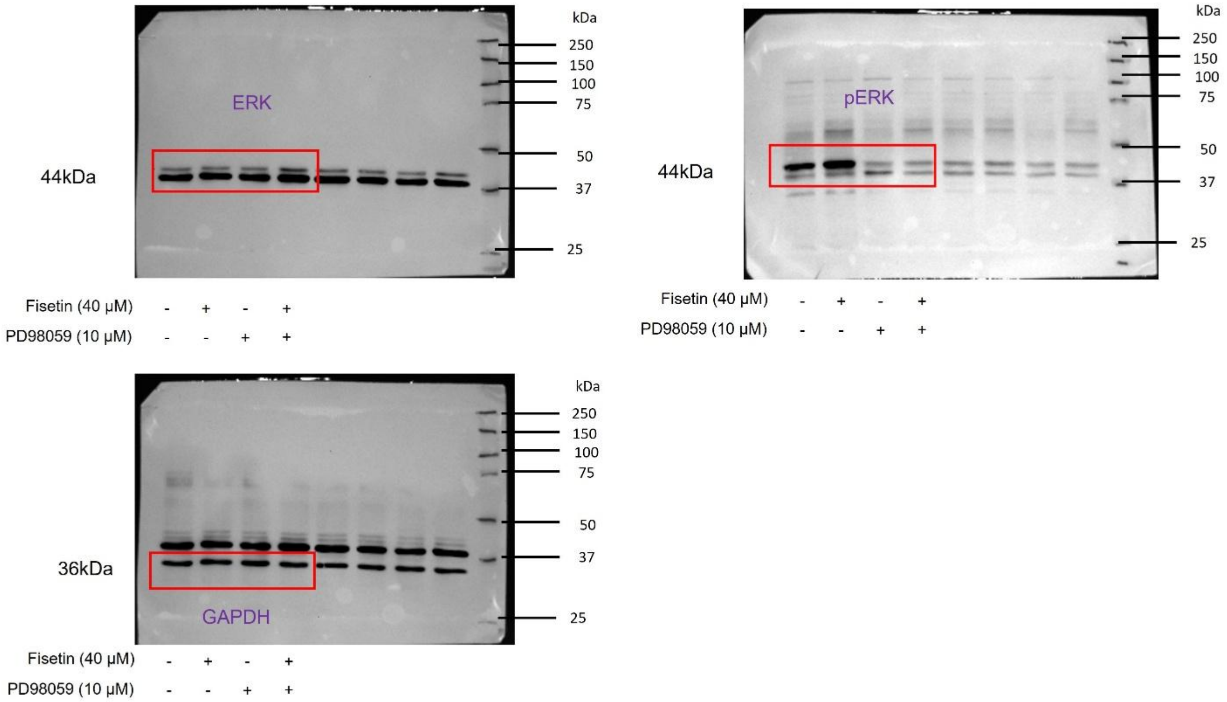

Figure S8. Uncropped gel images for Figure 5B

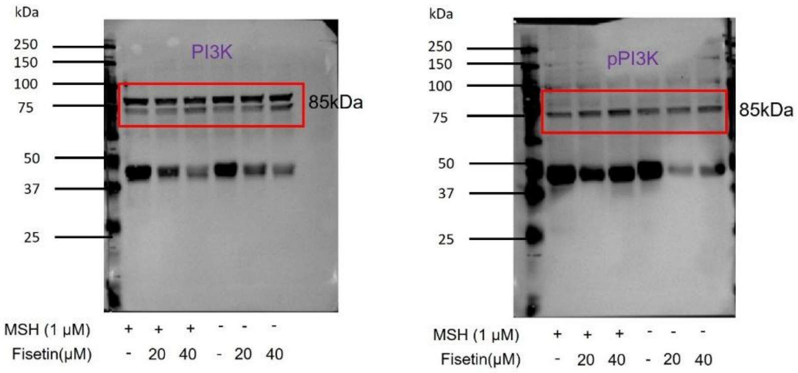

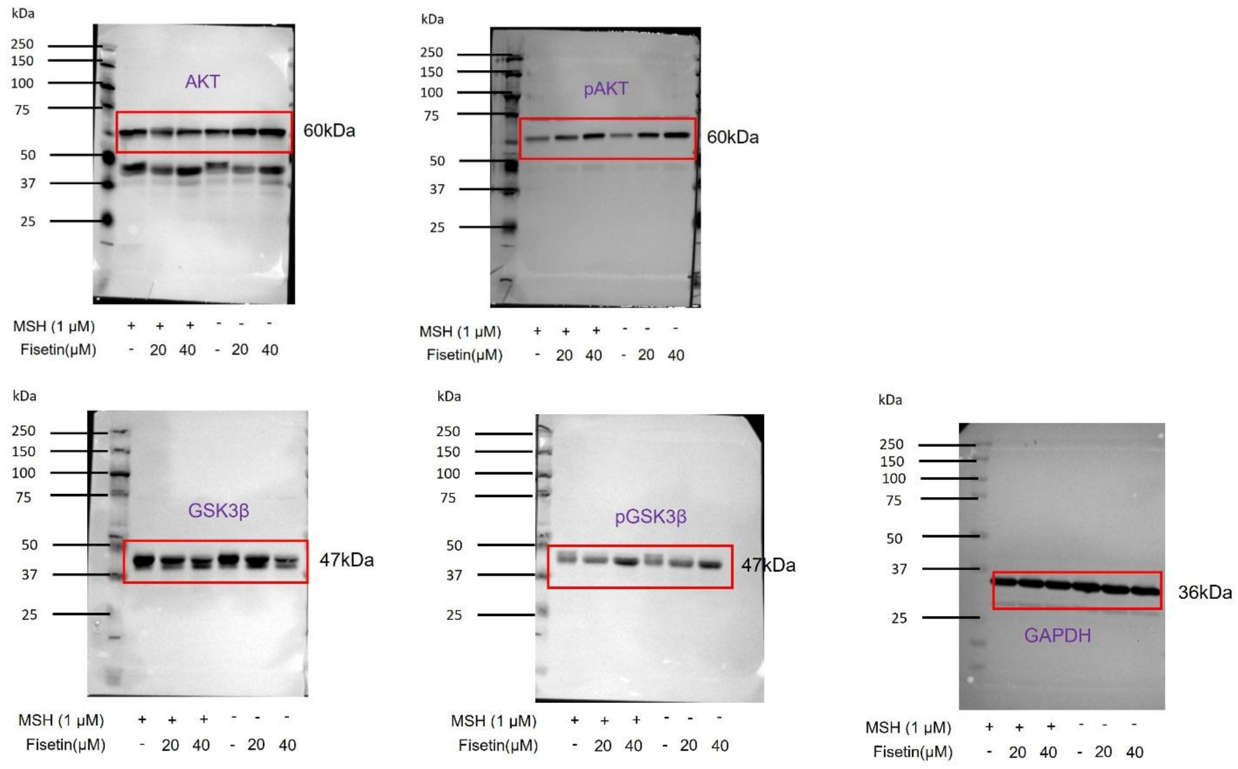

Figure S9. Uncropped gel images for Figure 5E

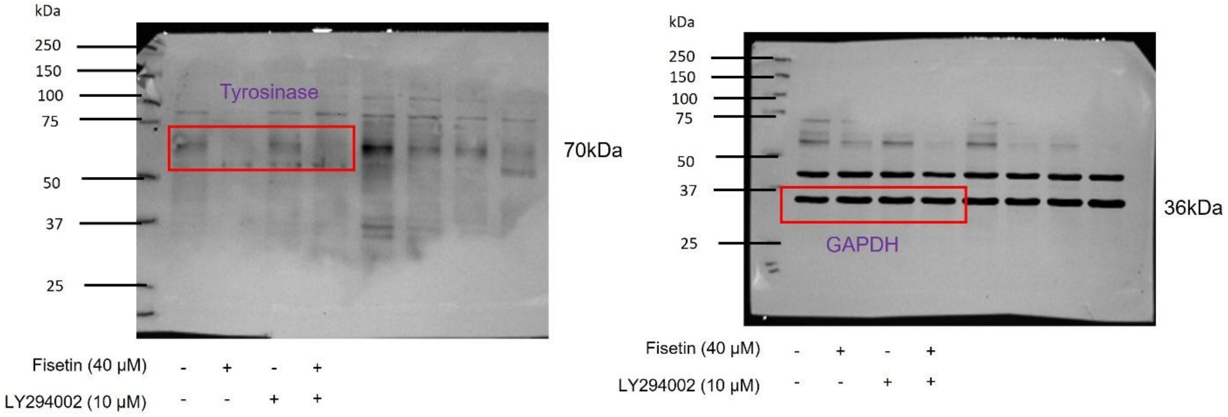

Figure S10. Uncropped gel images for Figure 5F

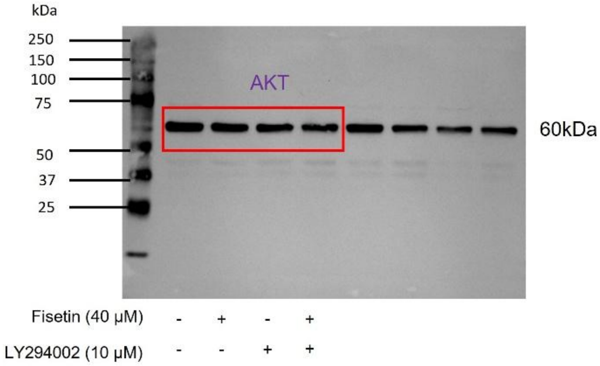

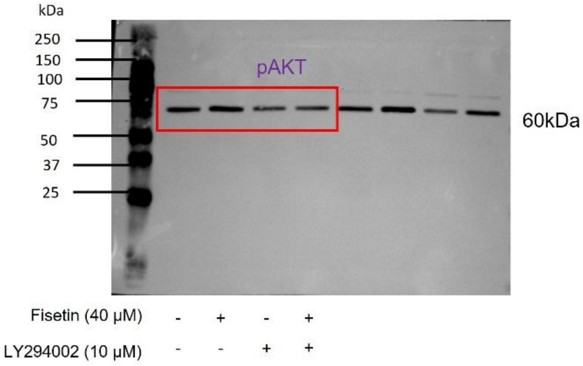

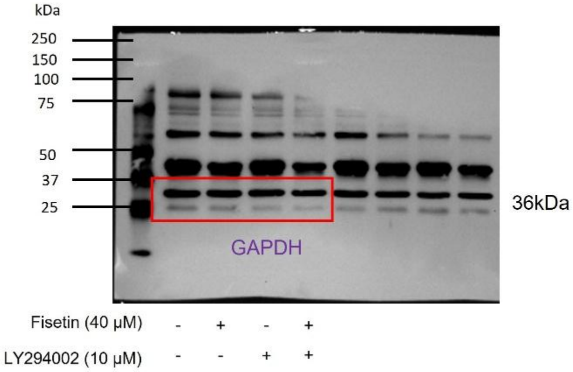

Supplement: Supplementary file 1 [file ijms-26-11739-s001.zip › ijms-3981658-supplementary.pdf]
